# Religion as a protective factor for health

**DOI:** 10.31744/einstein_journal/2020ED5562

**Published:** 2020-08-21

**Authors:** Marco Akerman, Rosilda Mendes, Samira Lima, Henrique Leonardo Guerra, Rafael Afonso da Silva, Daniele Pompei Sacardo, Juan Carlos Aneiros Fernandez

**Affiliations:** 1 Faculdade de Saúde Pública Universidade de São Paulo São PauloSP Brazil Faculdade de Saúde Pública, Universidade de São Paulo, São Paulo, SP, Brazil.; 2 Universidade Federal de São Paulo SantosSP Brazil Universidade Federal de São Paulo, Santos, SP, Brazil.; 3 Universidade Federal do Rio de Janeiro Rio de JaneiroRJ Brazil Universidade Federal do Rio de Janeiro, Rio de Janeiro, RJ, Brazil.; 4 Pontifícia Universidade Católica de Minas Gerais Belo HorizonteMG Brazil Pontifícia Universidade Católica de Minas Gerais, Belo Horizonte, MG, Brazil.; 5 Faculdade de Ciências Médicas Universidade Estadual de Campinas CampinasSP Brazil Faculdade de Ciências Médicas, Universidade Estadual de Campinas, Campinas, SP, Brazil.

The article “For an ´ecology of knowledge´ in health: an invitation from the *terreiros* to dialogue”,^(1)^brought many results of the research *Práticas Populares de Cuidado à Saúde em Comunidades de Terreiros* [Popular Health Care Practices in *Terreiro* Communities], carried out under the responsibility of the Center of Studies, Research, and Documentation in Healthy Cities (CEPEDOC), and financed by the Ministry of Health/Department of Strategic and Participatory Management/Department of Support to Participatory Management. The research was based upon several interactions among researchers and leaders of the African origin communities in 12 communities from four regions of the country, aiming to determine the existence of practices that generate and promote health in the *terreiros.* This perception was marked since the first case study conducted in 2014. The investigator’s report recorded the following definition of what health is for *candomblé* from Angola:

(...) it is very relative, a person may have a physical illness and be well, be healthy, because he/she is energetically very well, full of life, and there are people who have nothing physical, everything is functioning well, but he/she is ill, a person who is not well, is not happy, does not feel in harmony with the world, does not feel at peace with the world, cannot find their place in the world, and all this is the concept of health that goes from one extreme to the other. In the middle, there will be people who are physically ill, mentally ill, spiritually ill, and there will be those who are healthy in one area and unhealthy in another (...).

This statement can be associated with the relational/functional etiological model, as opposed to the ontological etiological model of disease characterizing healthcare practices in a general and broad manner, or, yet, as the health-disease *continuum* developed by Antonovsky,^(2)^ which equally, blurs the priority interest − if not exclusive − of disease and illness. Thus, diversity and singularity are acknowledged, with no moralization of ways of being in the world, and consequently, of producing health. This is because “there is no way to be healthy, to be hale and hearty, if the person feels violated.”

The theory of salutogenesis, developed by Silva et al.,^(1)^ was proposed as a possible approach for the epidemiological component, albeit with the bias of a study on health protective factors.

Hence, it focused on the field of health assets, with a perspective such as that adopted by the European Office of the World Health Organization (WHO). In this way, health assets “can operate on the person, group, community, and/or population levels as protective (or promoting) factors to cushion daily stress”.^(3)^ The expectation was that of “generating a salutogenic evidence base,”^(3)^so as to contribute to the:

discipline of modern epidemiology to move towards answering the question of what generates health, rather than the traditional approach of generating evidence on the causes and distribution of disease and early death.^(3)^

Both resilience and cohesion of a community, elements that are of interest to the health promotion approach, can be considered health assets.^(3)^In this text, we highlight the health asset called “Sense of Coherence”, as developed by Antonovsky.^(2)^

## SALUTOGENESIS AND SENSE OF COHERENCE

Salutogenesis is the approach proposed by Antonovsky^(2)^ to guide his investigation and the scientific development of the field of health promotion. Based on a critical evaluation of the development of this field – which, due to a lack of a consistent theoretical model with stated objectives, in general, adopts the paradigm of pathogenesis as reference –, a new model is proposed, focusing on elements that maintain and enhance health, that is, on the “health protective factors.”

This new orientation demands concepts and instruments that overcome the evaluation of health from its negative approach, that is, the disease. It would then be necessary to suggest new constructs and develop instruments that capture and quantify health-promoting aspects.

In this sense, Antonovsky^(2)^ presents a theoretical model, developed to improve the understanding about the relations between stressors, adaptation, and health. An operationalizing concept of this model is the Sense of Coherence (SOC), and its development resulted in the preparation of a 29-item scale, called Orientation to Life Questionnaire (OLQ).

Sense of Coherence is analyzed by means of three components: the stimuli arising from internal and external environments in the course of life are structured, predictable, and explainable; resources are available to meet the demands of these stimuli; and these demands are challenges, worthy of investment and engagement.^(2)^ These three components are known as comprehensibility, manageability, and meaningfulness.

The validity and reliability of SOC were evaluated by a systematic literature review of 458 scientific publications.^(4)^Sense of Coherence predicts positive events in long-term prospective studies, although there are divergent results, and proved to be reliable, valid, and applicable in different cultures as an instrument to measure how people manage stressful situations and keep themselves well.^(5)^

## THE RELIGIOUS EXPERIENCE AS HEALTH ASSET

### Religion and health

Since the 1980’s, studies have emerged, mainly in the United States, interested in seeking the influence of religious practice on people’s health.^(6)^Evidence of positive associations has been demonstrated, and there has been great repercussion in the press and academic media.^(6)^

These findings showed that people regularly attending religious services had lower rates of heart disease, cancer, hypertension, dementia, and symptoms, such as disability, depression, anxiety in the elderly, as well as mortality rates, than those who did not attend religious activities. These rates were 2,591/100 thousand for those who never attended compared to 1,308/100 thousand for those who attended churches once or more times a week.^(6)^

Many epidemiological findings at the beginning of these surveys even appear to be hyperbolic, since there are authors who claimed that the evidence of the presence of a religious factor in health is “overwhelming.”^(6)^

However, Levin et al.,^(7)^ showed there is not enough evidence to conclude that following a religion is positive and significantly related to health. Nevertheless, the authors present a theoretical basis for expecting such associations. By the way, Levin et al., was the first scientist to systematically review the empirical literature on religion and health, and the first scientist funded by the National Institutes of Health (NIH) to conduct research on the subject. He is the author of more than 175 academic publications, mainly on the instrumental functions of religion for physical and mental health and aging.

Despite the hyperboles, an epidemiological finding does not concern all *terreiro*-goers, but it means that in the “average,” or a frequency-based normality, religious involvement would be associated with lower morbidity and mortality rates.

Nowadays, more and more studies reiterate religion as a social determinant of health,^(8,9)^ but more research is still needed to translate this evidence into recommendations for individuals and society.

There is a lot of controversy about “how” and “why” attending religious activities would benefit people’s health. Certainly, epidemiology would not be the compass for this heuristic path, since it is only a method to raise some hypotheses of association.

Many studies^(5,6)^ venture to confront these doubts with explanations of the protective character of religions for health, such as the communitarian approach of religion, which strengthens bonds; the bias of religious beliefs as a manifestation of collective energies; the integration and high social cohesion of religious groups, which assure their members the most significant sense of certainty and common purposes of life - equated with Antonovski’s “sense of coherence”; social and existential support, a sense of purpose, a coherent system of beliefs, and a clear moral code provided by religion - although these can also come from other sources; the moral orientation of religions, which dictates behaviors in relation to the body and customs; the consideration of the body as sacred, present in many religions, and, finally, religious formulations about suffering, which help people deal with important losses and hopelessness.^(6)^

Attending religious activities is not directly linked to the intensity of devotion, and institutionalized religiosity is not always related to spiritual experience.^(5)^ This is challenging in the case of *candomblé* followers, who observe cautious rituals in all contexts of their own life experience, and for some of these practitioners, religion is even confused with a civilizing process.

There are no reports of international data analyzing the influence of African religions on people’s health. There are studies in English that use the terms “church-goers” to qualify the subjects of the research, which are implied to be Christian churchgoers.^(10)^

In Brazil, the relation between religion and health has been studied and presented for discussion from different perspectives. Some studies have been concerned with investigating the relations between religiosity and improvements, in order to confront existential losses of different nature.^(11,12)^

### Sense of Coherence, Health, and Religion

In a systematic literature review, Eriksson et al.,^(4)^ observed that SOC is strongly related to health perception, especially mental health. The higher the SOC score, the better the overall perceived health, at least for those with an initial high SOC. This relation occurs in study populations regardless of age, sex, ethnicity, nationality, and study design. Sense of Coherence seems to play an important role in moderating or mediating the health explanation. In addition, it must be able to predict health. It is an important contributor to the development and maintenance of people’s health, but it cannot explain health in general in isolation, and is a health-promoting resource that strengthens resilience and develops a positive subjective state of health. Hence, salutogenesis becomes a valuable approach to health promotion and should be implemented more frequently.

In a population-based study conducted in Finland, Eriksson et al.,^(13)^ using the short version (13 items) of the Antonovsky’s Sense of Coherence scale, observed that the SOC was significant and strongly related to the health self-perception score, providing evidence of the potential of the SOC concept as a positive indicator of mental health, then taken as a health promotion resource that supports the development of a positive subjective health status.

## CONCLUSION

Escaping from the paradigm of pathogenesis or defect as the focus of health work has been the desideratum of theories that seek to screen protective factors or social determinants as the raw material of a more critical approach to health promotion.

For this, we brought the concept of “salutogenesis,” which is dedicated to think of the production of health as the capacity of subjects to work on health through the articulation of their understanding, their management, and the production of meanings of different vital events they experience.

The main interest should not be to make associations that search for evidence on the causes of the diseases, but to point out the attributes of exposure as analyzer of the outcome (generate more health).

This positive perspective of exposure and outcome in epidemiology would open ways to explore more widely how and why attending religious activities would benefit people’s health.
