# Supplementary material for: Religion as a protective factor for health
Source: Einstein (Sao Paulo). 2020 Aug 21;18:eED5562. doi: 10.31744/einstein_journal/2020ED5562 (PMC7444599; doi:10.31744/einstein_journal/2020ED5562)
Supplement: Translation

# Translation: Religião como fator protetor para saúde

**Authors:** Marco Akerman, Rosilda Mendes, Samira Lima, Henrique Leonardo Guerra, Rafael Afonso da Silva, Daniele Pompei Sacardo, Juan Carlos Aneiros Fernandez

**Affiliations:** 1 Brasil Faculdade de Saúde Pública, Universidade de São Paulo, São Paulo, SP, Brasil.; 2 Brasil Universidade Federal de São Paulo, Santos, SP, Brasil.; 3 Brasil Universidade Federal do Rio de Janeiro, Rio de Janeiro, RJ, Brasil.; 4 Brasil Pontifícia Universidade Católica de Minas Gerais, Belo Horizonte, MG, Brasil.; 5 Brasil Faculdade de Ciências Médicas, Universidade Estadual de Campinas, Campinas, SP, Brasil.

O artigo “Para uma ‘ecologia de saberes’ em saúde: um convite dos terreiros ao diálogo”^(1)^ traz resultados da pesquisa Práticas Populares de Cuidado à Saúde em Comunidades de Terreiros, realizada sob responsabilidade do Centro de Estudos, Pesquisa e Documentação em Cidades Saudáveis (CEPEDOC), com financiamento do Ministério da Saúde/Secretaria de Gestão Estratégica e Participativa/Departamento de Apoio à Gestão Participativa. A pesquisa foi construída em interações diversas entre pesquisadores e lideranças de comunidades de matriz africana em 12 comunidades de quatro regiões do país para delinear a existência de práticas produtoras e promotoras de saúde no interior dos terreiros. Essa percepção ficou marcada já no primeiro estudo de caso realizado em meados de 2014. O relatório do pesquisador registrou a seguinte definição do que é a saúde para o candomblé de Angola:

(...) é muito relativo, a pessoa pode ter uma doença física e estar bem, ser saudável, porque ela está energeticamente bem, está plena, e tem gente que não tem nada físico, está tudo funcionando fisiologicamente perfeito, mas ela está doente, é uma pessoa que não está bem, não é feliz, não se sente em harmonia com o mundo, não se sente em paz com o mundo, não acha lugar para ela no mundo, tudo isso é conceito de saúde que vai passar de um extremo para o outro, nesse meio vai ter gente doente fisicamente, doente mentalmente, espiritualmente, vai ter gente saudável numa área e não saudável em outra (...).

Essa afirmação pode ser associada ao modelo etiológico relacional/funcional, em oposição ao modelo etiológico ontológico da doença, que caracteriza as práticas em saúde de modo geral e amplo, ou, ainda ao *continuum* saúde-doença desenvolvido por Antonovsky,^(2)^ que, igualmente, desfoca o interesse prioritário − senão exclusivo − da doença e do adoecer. Reconhecem-se, assim, a diversidade e a singularidade desprovidas de uma moralização dos modos de ser e estar no mundo − e, consequentemente, de produzir saúde. Isso porque “não tem como ter saúde, não tem como ser saudável se a pessoa se sentir violada”.

A teoria da salutogênese, desenvolvida por Silva et al.,^(1)^ foi proposta como possível abordagem para o componente epidemiológico, mas com o viés de um estudo sobre os fatores protetores da saúde.

Adentrou-se, assim, no campo dos ativos em saúde, em uma perspectiva como a adotada pelo escritório europeu da Organização Mundial da Saúde (OMS). Dessa forma, os ativos em saúde “podem operar a nível de pessoa, grupo, comunidade e/ou população como fatores de proteção (ou promoção) para amortecer o estresse diário”.^(3)^A expectativa era a de se “gerar uma base de evidência ‘salutogênica’”,^(3)^ de modo a contribuir para que a:

disciplina da epidemiologia moderna se mova em direção à busca de resposta à pergunta sobre o que gera saúde, mais do que em direção ao enfoque tradicional de geração de evidência sobre as causas e distribuição da doença e da morte precoce.^(3)^

Tanto a resiliência quanto a coesão de uma comunidade, elementos que interessam à abordagem da promoção da saúde, podem ser consideradas ativos em saúde.^(3)^Nesse texto, damos destaque ao ativo em saúde denominado “Senso de Coerência”, como desenvolvido por Antonovsky.^(2)^

## SALUTOGÊNESE E SENSO DE COERÊNCIA

A salutogênese é a abordagem proposta por Antonovsky^(2)^ para guiar sua pesquisa e o desenvolvimento científico do campo da promoção da saúde. A partir de uma avaliação crítica quanto ao desenvolvimento desse campo – que, por carecer de um modelo teórico consistente com seus objetivos declarados, adota, em geral, o paradigma da patogenia como referência –, é proposto um novo modelo, que se concentra em elementos que mantêm e aprimoram a saúde, ou seja, nos “fatores protetores da saúde”.

Essa nova orientação demanda conceitos e instrumentos que superem a avaliação da saúde, a partir de seu negativo − a doença. Seria necessário, então, sugerir novos construtos e desenvolver instrumentos que captem e quantifiquem aspectos promotores de saúde.

Nesse sentido, Antonovsky^(2)^ apresenta um modelo teórico, desenvolvido para aprimorar o entendimento sobre as relações entre estressores, adaptação e saúde. Um conceito operacionalizador desse modelo é o Senso de Coerência (SOC), e seu desenvolvimento resultou na construção de uma escala de 29 itens, chamada *Orientation to Life Questionnaire* (OLQ).

O SOC é analisado por meio de três componentes: os estímulos decorrentes dos ambientes internos e externos no curso de vida são estruturados, previsíveis e explicáveis; os recursos estão disponíveis para atender às demandas desses estímulos; e essas demandas são desafios, dignos de investimento e engajamento.^(2)^ Esses três componentes são conhecidos como: compreensão (*comprehensibility*), manejo (*manageability*) e sentido (*meaningfulness*).

A validade e a confiabilidade do SOC foram avaliadas por revisão sistemática da literatura envolvendo 458 publicações científicas.^(4)^ O SOC prediz eventos positivos em estudos prospectivos de longa duração, embora existam resultados divergentes, e mostra-se confiável, válido e aplicável em diferentes culturas, como instrumento de medição de como as pessoas manejam situações de estresse e mantêm-se bem.^(5)^

## A EXPERIÊNCIA RELIGIOSA COMO ATIVO EM SAÚDE

### Religião e saúde

A partir da década de 1980, surgiram estudos, principalmente nos Estados Unidos, interessados em buscar influências da prática religiosa na saúde das pessoas.^(6)^ Foram demonstradas evidências de associações positivas, e a repercussão foi grande na imprensa e nos meios acadêmicos.^(6)^

Esses achados mostraram que pessoas assistindo regularmente a serviços religiosos apresentaram taxas mais baixas relacionadas a doenças cardíacas, câncer, hipertensão, demência e de sintomas como invalidez, depressão, ansiedade em idosos, além de mortalidade, do que as que não frequentavam atividades religiosas. Essas taxas eram de 2.591/100 mil para aqueles que nunca frequentaram em comparação a 1.308/100 mil para aqueles que frequentaram igrejas uma ou mais vezes por semana.^(6)^

Muitos achados epidemiológicos no início dessas pesquisas chegam a parecer hiperbólicos, pois há autores que afirmavam que as evidências da presença de um fator religioso na saúde são “esmagadoras”.^(6)^

Entretanto, Levin et al.,^(7)^ mostram que não há evidências suficientes para concluir que seguir uma religião está positivo e significativamente relacionado à saúde. Os autores apresentam, ainda assim, uma base teórica para esperar tais associações. A propósito, Levin et al., foi o primeiro cientista a revisar sistematicamente a literatura empírica sobre religião e saúde e o primeiro cientista financiado pelo *National Institutes of Health* (NIH) a realizar pesquisas sobre o tema. É autor de mais de 175 publicações acadêmicas, principalmente sobre as funções instrumentais da religião para a saúde física e mental e o envelhecimento.

Não obstante as hipérboles, um achado epidemiológico não diz respeito a todos os frequentadores, mas significa que, na “média”, ou em uma normalidade baseada em frequências, o envolvimento religioso estaria associado com taxas menores de morbidade e mortalidade.

Atualmente, cada vez mais estudos reiteram a religião como determinante social da saúde,^(8,9)^ mas ainda são necessárias mais pesquisas para traduzir essas evidências em recomendações para indivíduos e a sociedade.

Há muita controvérsia sobre “como” e o “por que” frequentar atividades religiosas beneficiariam a saúde das pessoas. Certamente, a epidemiologia não seria a bússola para esse caminho heurístico, pois constitui apenas um método para levantar algumas hipóteses de associação.

Muitas pesquisas^(5,6)^ aventuram-se a enfrentar essas dúvidas com explicações do caráter protetivo das religiões para a saúde, como a abordagem comunitária da religião, que fortalece vínculos; o viés das crenças religiosas como manifestação de energias coletivas; a integração e a alta coesão social dos grupos religiosos, que asseguram a seus membros o senso de certeza e os propósitos comuns de vida mais significativos − equiparados ao “sentido de coerência” de Antonovski; o apoio social e existencial, o senso de propósito, um sistema coerente de crenças e um claro código moral fornecidos pela religião − embora estes também possam vir de outras fontes; a orientação moral das religiões, que dita comportamentos em relação ao corpo e aos costumes; a consideração do corpo como sagrado, presente em muitas religiões, e, por fim, as formulações religiosas sobre o sofrimento, que ajudam as pessoas a lidarem com as perdas importantes e a desesperança.^(6)^

A frequência em atividades religiosas não está diretamente ligada à intensidade da devoção, e nem sempre a religiosidade institucionalizada está relacionada com experiência espiritual.^(5)^ Isso é desafiador no caso dos praticantes do candomblé, que observam cautelas rituais em todos os contextos da própria experiência de vida, e, para uma parte desses praticantes, a religião confunde-se mesmo com um processo civilizatório.

Não se têm notícias de dados internacionais que tenham analisado a influência de religiões de matriz africana na saúde das pessoas. Existem estudos em língua inglesa que usam os termos “*church-goers*” para qualificar os sujeitos das pesquisas, os quais ficam subentendidos se tratar de frequentadores de igrejas cristãs.^(10)^

No Brasil, as relações entre religião e saúde vêm sendo estudadas e apresentadas para discussão de diferentes perspectivas. Alguns estudos têm se preocupado em investigar as relações entre religiosidade e melhorias, para o enfrentamento de perdas existenciais de natureza diversa.^(11,12)^

### Senso de Coerência, Saúde e Religião

Em revisão sistemática da literatura, Eriksson et al.,^(4)^ observaram que o SOC está fortemente relacionado à percepção de saúde, especialmente da saúde mental. Quanto mais forte o SOC, melhor a saúde percebida em geral, pelo menos para aqueles com um alto SOC inicial. Essa relação ocorre em populações de estudo, independentemente de idade, sexo, etnia, nacionalidade e do desenho do estudo. O SOC parece ter importante papel de moderar ou mediar a explicação da saúde. Além disso, ele deve ser capaz de prever a saúde. Trata-se de importante contribuinte para o desenvolvimento e a manutenção da saúde das pessoas, mas não pode explicar, isoladamente, a saúde em geral, constituindo um recurso de promoção da saúde, que fortalece a resiliência e desenvolve um estado subjetivo positivo de saúde. Assim, torna-se a salutogênese uma abordagem valiosa para a promoção da saúde, devendo ser implementada com maior frequência.

Em estudo de base populacional conduzido na Finlândia, Eriksson et al.,^(13)^ utilizando a versão curta (13 itens) do Questionário de Senso de Coerência de Antonovsky (QSCA), observaram que o SOC foi significativo e esteve fortemente relacionado com a pontuação de autopercepção de saúde, fornecendo evidência do potencial do conceito do SOC como indicador positivo de saúde mental, tomado, então, como um recurso de promoção da saúde, que apoia o desenvolvimento de um estado subjetivo positivo de saúde.

## CONCLUSÃO

Escapar do paradigma da patogenia ou do defeito como foco principal do trabalho em saúde tem sido o desiderato das teorias que buscam colocar em tela os fatores de proteção ou os determinantes sociais como matéria-prima de uma abordagem mais crítica da promoção da saúde.

Para tal, trouxemos o conceito de “salutogênese”, que se dedica a pensar a produção da saúde como a capacidade de os sujeitos trabalharem a saúde por meio de articulação de sua compreensão, seu manejo e da produção de sentidos de distintos eventos vitais que experimentam.

O interesse maior não deveria ser fazer associações que busquem evidências sobre as causas das doenças, mas apontar atributos da exposição como analisador do desfecho (gerar mais saúde).

Essa perspectiva positiva da exposição e do desfecho em epidemiologia abriria caminhos para se explorarem, de maneira mais ampliada, o como e o por que frequentar atividades religiosas beneficiaria a saúde das pessoas.
